# Logistic Regression for Machine Learning in Process Tomography

**DOI:** 10.3390/s19153400

**Published:** 2019-08-02

**Authors:** Tomasz Rymarczyk, Edward Kozłowski, Grzegorz Kłosowski, Konrad Niderla

**Affiliations:** 1 Research & Development Centre Netrix S.A., University of Economics and Innovation in Lublin, 20-209 Lublin, Poland; 2 Faculty of Management, Lublin University of Technology, 20-618 Lublin, Poland

**Keywords:** machine learning, electrical impedance tomography, ultrasound tomography, process tomography, image reconstruction

## Abstract

The main goal of the research presented in this paper was to develop a refined machine learning algorithm for industrial tomography applications. The article presents algorithms based on logistic regression in relation to image reconstruction using electrical impedance tomography (EIT) and ultrasound transmission tomography (UST). The test object was a tank filled with water in which reconstructed objects were placed. For both EIT and UST, a novel approach was used in which each pixel of the output image was reconstructed by a separately trained prediction system. Therefore, it was necessary to use many predictive systems whose number corresponds to the number of pixels of the output image. Thanks to this approach the under-completed problem was changed to an over-completed one. To reduce the number of predictors in logistic regression by removing irrelevant and mutually correlated entries, the elastic net method was used. The developed algorithm that reconstructs images pixel-by-pixel is insensitive to the shape, number and position of the reconstructed objects. In order to assess the quality of mappings obtained thanks to the new algorithm, appropriate metrics were used: compatibility ratio (CR) and relative error (RE). The obtained results enabled the assessment of the usefulness of logistic regression in the reconstruction of EIT and UST images.

## 1. Introduction

Tomography is a non-invasive method of identifying the interior of objects [1]. The non-destructive nature of this method is both its advantage and disadvantage. Lack of the necessity of damaging or total destruction of the examined object is burdened with the necessity to solve the inverse problem, which is an immanent feature of every type of tomography [2,3,4]. 

The inverse problem belongs to the group of so-called ill-posed problems. According to Hadamard, well-conditioned problems must meet three criteria: the solution exists, the solution is unambiguous and the solution is stable [5]. A well-posed problem is likely to be solved on a computer using a stable algorithm [6,7,8]. If the problem is not well-posed, it should be reformulated in a way that allows the use of a numeric algorithm [9]. In such cases, additional assumptions are usually applied, e.g., a smoothness of the solution. Such a process is called regularization. For example, regularization of linear problems is usually carried out by Tikhonov regularization.

In the monitoring of industrial processes (e.g., during crystallization), several measuring techniques can be used simultaneously. One of the possibilities is to combine two methods—electrical impedance tomography and ultrasound transmission tomography. The physical source of data may be electricity, magnetism, sound waves, electromagnetic waves, X-rays, visible light, etc. The most common types of tomography used in industrial processes and medicine are: electrical impedance tomography (EIT) [10,11,12], computed tomography (CT) [13], radio tomographic imaging (RTI) [14], electrical capacitance tomography (ECT) [15,16,17,18,19,20] and ultrasound transmission tomography (UST) [21].

EIT is included in a wider group of methods referred to as electrical tomography (ET) [22,23,24]. ET involves several techniques for image reconstruction including: EIT [25], ERT which is a variation of EIT, and ECT [15,18,19,26,27]. In ECT, we determine the value of the electrical permittivity ε [28,29,30], while EIT and ERT reconstruct the conductivity σ [31]. The difference between EIT and ERT is mainly a matter of name. The term ERT is usually used when the nature of the studied environment is evidently resistant [32]. This is the case, for example, in geological applications of tomography [33,34].

The EIT tomography includes, for example, process industrial tomography, which consists of non-invasive methods of imaging the interior of tanks, reactors or pipes. While the typical area of CT applications is medicine, ERT tomography is applied in the study of geology, and EIT tomography is applied in industry [35]. UST is associated with some forms of medical research, while in industry this method is still not widely used. This is confirmed by the quotation from [36], in which the authors write “in oil and gas industry, the applications of ultrasonic tomography technique for flaw detection in pipeline are limited as this technology is still in its infancy”. When comparing electrical, magnetic and ultrasound tomography, it can be noted that the most innovation is in the field of electrical impedance tomography (EIT) [35], electrical capacitance tomography (ECT) [26] and magnetic resonance [37,38]. 

There are certain technical and construction conditions of process industrial facilities, which mean the EIT method cannot be used. This happens if, for example, the interior environment of the reactor or tank is highly dielectrical, or the walls of the tank conduct electricity and, due to moisture, the insulation of the electrodes becomes very difficult. In these situations, the appropriate method to replace EIT is UST.

Among the existing problems of process tomography, the following topics can be mentioned: corrosion control of wall loss in places of pipe support [39], imaging of hidden defects inside metal elements [40], reinforced concrete inspection [23,41], detection of cracks or voids in nonmetallic materials [42], monitoring flaws of gas pipelines [36], bubble detection for two-phase liquid and gas [15,20,43], monitoring of flood embankments [11,34,44] and others.

The general purpose of using tomography is a non-invasive imaging of the object interior with the highest possible accuracy and speed. This goal is particularly difficult to achieve if the inclusions are relatively small and occur individually (not in clusters). To overcome these problems, attempts are made to optimally select physical and computational methods for a specific type of tested objects. Researchers are striving to develop a tomography scanner that can be applied to the widest possible range of problems.

The main goal of the research presented in this article is to develop a refined machine learning algorithm for industrial tomography applications. The method proposed in this study is based on machine learning linked to logistic regression. In the literature, one can find attempts to apply logistic regression in both process and medical tomography [45,46].

Most of the novelties in tomographic issues include both equipment (sensors, computer systems) as well as algorithms solving the inverse problem. Considering the frequency and the number of new scientific achievements regarding innovative process industrial solutions, UST is less developed than other tomographic methods. This may be due to the difficulty of developing an efficient system that includes transducers and algorithms that would enable accurate image reconstruction under industrial conditions.

The classic way of solving the inverse problem in tomography is Gauss-Newton method. Other than this approach, currently the most commonly used methods employ machine learning algorithms [27,44]. The popular methods of machine learning used in tomography include neural networks [31], deep learning [27] and statistical methods [47]. Logistic regression is counted among statistical methods of machine learning. Due to the original structure, the presented algorithm based on logistic regression is a fresh proposition in the field of process tomography. Additionally, in order to solve the problem of selecting the relevant input variables in the regression model, the elastic net method was used.

This article consists of four sections. Section 1 presents the state of art regarding tomographic methods and algorithms used in the reconstruction of images in process tomography. Many types of tomography are discussed, taking into account their most common practical applications. A detailed description of the scientific contribution and novelties contained in the presented concept can be found in Section 2. This section also contains a description of the test stand, the data used, elastic net regularization, the multiple logistic regression system (LRS) concept and algorithm, as well as information on the machine learning process. Section 3 presents examples of reconstructions obtained by using the multiple LRS method. The quality of the logistic regression for EIT and UST is also estimated. Two indicators are used as measures of image reconstruction quality: compatibility ratio (CR) and relative error (RE). Section 4 contains a summary, conclusions and directions for further work.

## 2. Materials and Methods

The article presents two types of tomography—EIT and UST. Each type of tomography requires the use of different hardware. The differences concern sensors and equipment for displaying the output image. In EIT, images are created on a mesh of triangular pixels with a resolution of 2883. UST uses a square screen with a resolution of 128 × 128, which gives 16,384 pixels.

**Figure 1:**
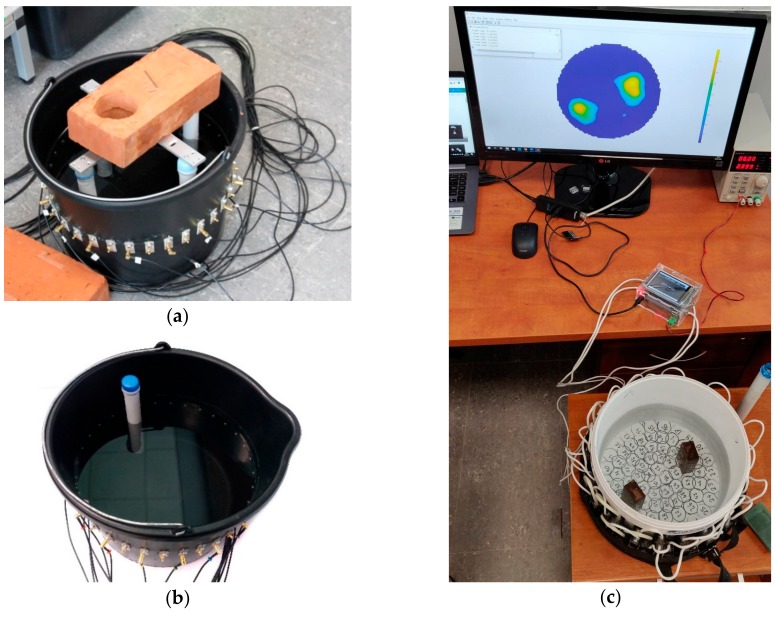
The physical model of the reactor with: (**a**,**b**) electrical impedance tomography (EIT) electrodes, (**c**) ultrasound transmission tomography (UST) transducers.

A common feature for both methods is the application of a machine learning algorithm that uses logistic regression to solve the inverse problem, and elastic net regularization of input values. Figure 1 presents test benches for the EIT and UST methods. Based on the measurements carried out, an algorithm was developed to generate training cases necessary for machine learning using computer simulation. The validation of the algorithm was made by comparing simulated measurements with real measurements.

### 2.1. The Novelty of the Presented Solution

The novelty of the algorithm presented in this article is the combination of elastic net methods and logistic regression to generate the output image from the examples of EIT and UST. The original approach is to use an algorithm consisting of many trained subsystems of an elastic net and logistic regression system (LRS). An elastic net is used to reduce the vector of input variables by removing mutually correlated predictors. After transforming predictors into pixel binary values, a tomographic image is generated. In the case of EIT, the input data for LRS are measurements of voltage drops between the electrodes. For UST, the predictors are the velocity of ultrasound waves emitted in the examined cross-section of the object. The use of multiple LRS generating single-pixel binary classifiers instead of a complex system generating multiple pixels of the output image increases the accuracy of the reconstruction.

**Figure 2:**
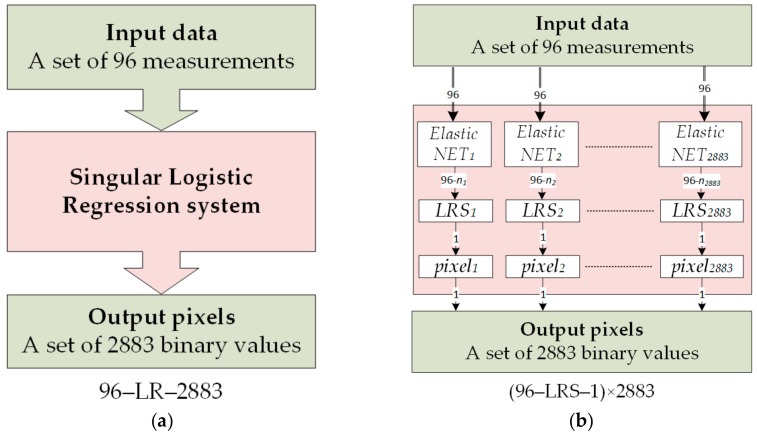
Comparison of algorithms for: (**a**)—single logistic regression unit, (**b**)—multiple LRS.

The originality of the algorithm is analogous in both the EIT and UST applications. Figure 2a concerns the EIT. It shows an algorithm based on the structure of an ordinary, singular logistic regression unit with 96 inputs and 2883 binary {0,1} outputs. In Figure 2b, for comparison, the multiple LRS with elastic nets scheme is shown. Each of the 2883 LRS after the input data reduction using elastic nets generates a binary classifier as the output, which is a pixel value. Then, reconstructions of all 2883 pixels make up the output image. In the case of UST, the workflow is the same. Only the number of inputs and the resolution of the reconstructed image change.

### 2.2. Logistic Regression

The main goal of both electric and ultrasound tomography is reconstruction of the cross-section called the field of view. In many cases, we need to specify cross-sections of areas where there are hidden objects requiring imaging. To identify these inclusions, the imaging domain was first defined as a specially developed pixel mesh, acting as finite elements [48]. In the case of EIT, it was a mesh of triangular elements, and for UST they were squares. To answer the question of whether a finite element contains inclusion, it was necessary to define a function whose results belong to a set of binary values {0,1}. The results of this function identify the object by selecting the appropriate pixel with a different color than the background.

In logistic regression, we calculate the probability that the realization of the output variable belongs to the appropriate category. In this case the probability of inclusion was estimated. In addition, the use of logistic regression allows the determination of the resolution of the imaging domain.

Let us consider a data set in which the implementation of the response variable belongs to a binary file. For each finite element, we analyze the training set $D={\left\{\left(x_{i},y_{i}\right)\right\}}_{1⩽i⩽n}$, where ${\left\{x_{i}\right\}}_{1⩽i⩽n}$ means a series of input variables, ${\left\{y_{i}\right\}}_{1⩽i⩽n}$ is a vector of response variable, and $\;x_{i}\in R^{m}$, $y_{i}\in \left\{0,1\right\}$ for 1⩽i⩽n and m denotes number of measurements gained from transducers and sensors. If the finite element reflects inclusion, then we assume $y_{i}=1$ otherwise we take $y_{i}=0$. The training set can be described as $D=\left\{Y,X\right\}$, where

$$Y=\left[\begin{array}{c} y_{1}\\ y_{2}\\ \vdots \\ y_{n} \end{array}\right],\;X=\left[\begin{array}{cccc} x_{11} & x_{12} & \cdots & x_{1m}\\ x_{21} & x_{22} & \cdots & x_{2m}\\ \vdots & \vdots & \vdots & \vdots \\ x_{n1} & x_{n2} & \cdots & x_{nm} \end{array}\right]=\left[\begin{array}{c} x_{\left(1\right)}\\ x_{\left(2\right)}\\ \vdots \\ x_{\left(n\right)} \end{array}\right]$$

Detecting the signal $x_{i}\in R^{m}$ gained from sensors or transducers, it is obligatory to classify the presence or not of inclusion in the finite element. The objective is to discover such a classifier $f:R^{m}\to \left\{0,1\right\}$, which allows categorization of the presence of object into categories y=1 or y=0 based on observation $x\in R^{m}$.

We define a random variable Y with binomial distribution, i.e., $Y:\Omega \to \left\{0,1\right\}$ on probability space $\left(\Omega ,F,P\right)$. Logistic regression is a method in which the Y response variable has a binomial distribution. Based on observation of input variables X the logistic regression [49,50,51] describes probability of realization of dependent variable Y. Therefore, it is necessary to determine probabilities of success $P\left(Y=1|X\right)$, and defeat $P\left(Y=0|X\right)$. In the literature the odds are defined as in Equation (1):

(1) $$\Theta \left(X\right)\;=\;\frac{P\left(Y=1\left|X\right.\right)}{P\left(Y=0\left|X\right.\right)}\;=\;\frac{P\left(Y=1|X\right)}{1-P\left(Y=1|X\right)}$$

Thus, the odds are the ratio probability of success to probability of defeat. The objective of logistic regression is to determine the probability of success $p\left(X\right)=P\left(Y=1|X\right)$ based on observation X. Since the probability of success $p\left(X\right)\in \left(0,1\right)$, based on Equation (1) it results that the odds $\Theta \left(X\right)\in \left(0,\infty \right)$ but $ln\left(\Theta \left(X\right)\right)\in \left(-\infty ,\infty \right)$. The logarithm of odds can be called log-odds or logit. In logistic regression the linear dependencies between log-odds and input variables are analyzed as in Equation (2):

(2) $$ln\Theta \left(X\right)=ln\left(\frac{p\left(\beta ,X\right)}{1-p\left(\beta ,X\right)}\right)=X\beta$$

where $\beta =\left(\beta_{1},\ldots ,\beta_{m}\right)\in R^{m}$. In the case that linear Equation (2) has an intercept, then the column that corresponds to the intercept in matrix X contains ones. From Equation (2) we derive Equation (3):

(3) $$p\left(\beta ,X\right)=\frac{e^{X\beta }}{1-e^{X\beta }}$$

Generally, to estimate the unknown parameters β in Equation (3), the maximum likelihood technique is applied. From above, the task shown in Equation (4)

(4) $$\underset{\beta }{max}\;L\left(\beta ,Y,X\right)$$

must be solved, where the likelihood function is defined as in Equation (5):

(5) $$L\left(\beta ,Y,X\right)=\overset{n}{\underset{i=1}{\prod }}p{\left(\beta ,x_{\left(i\right)}\right)}^{y_{i}}{\left(1-p\left(\beta ,x_{\left(i\right)}\right)\right)}^{1-y_{i}}$$

As a replacement for solving Equation (4) we solve the auxiliary task shown in Equation (6):

(6) $$\underset{\beta }{max}\;l\left(\beta ,Y,X\right)$$

where the objective function is defined as the logarithm of the likelihood function $l\left(\beta ,Y,X\right)=ln\left(L\left(\beta ,Y,X\right)\right)$ and equals Equation (7):

(7) $$l\left(\beta ,Y,X\right)=\overset{n}{\underset{i=1}{\sum }}\left(y_{i}x_{\left(i\right)}\beta -ln\left(1\;+\;e^{x_{\left(i\right)}\beta }\right)\right)$$

The Newton–Raphson algorithm was applied to determine the unknown parameters β. Submission of this algorithm reasons that the unknown parameters β are estimated by iterative steps. In the step j + 1 the estimators are determined by Equation (8):

(8) $$\beta_{j\;+\;1}=\beta_{j}\;+\;{\left(\frac{\partial^{2}l}{\partial \beta \partial \beta^{T}}\left(\beta_{j}\right)\right)}^{-1}\frac{\partial l}{\partial \beta }\left(\beta_{j}\right)$$

where $\frac{\partial l}{\partial \beta }\left(\beta \right)$, $\frac{\partial^{2}l}{\partial \beta \partial \beta^{T}}\left(\beta \right)$ represent first and second partial derivatives of the objective function (7).

### 2.3. Elastic Net

Taking into account the measurements obtained from individual electrode pairs and transducers, one can note that the values are strongly correlated. This phenomenon is referred to as the problem of multicollinearity. If the independent variables (predictors) in the system shown in Equation (2) are correlated, the direct solution of the task in Equation (6) with the direct application of the Newton–Raphson algorithm does not give the expected result. An additional problem is the lack of stability of forecasts based on this model. Therefore, from the full measurement vector, the appropriate predictors (input variables) should be selected, which will then be included in the regression model of Equation (2). Selected predictors should significantly affect the response values and, at the same time, should not generate multicollinearity. 

There are many techniques to solve the optimization problem of input variable correlation (6). Among these can be mentioned singular value decomposition, regularization or least angle regression. In the literature, proper examples can be found in [50,52]. A possible way to reduce the problem of multicollinearity between predictors is the application of the elastic net method [49]. An elastic net relies on enforcing a penalty on large values of estimators and involving this penalty in the objective function. To determine the unknown parameters of the logistic regression in Equation (2) for correlated predictors we should solve the task of Equation (9):

(9) $$\underset{\beta }{max}\overset{n}{\underset{i=1}{\sum }}\left(y_{i}x_{\left(i\right)}\beta -ln\left(1\;+\;e^{x_{\left(i\right)}\beta }\right)\right)-\lambda P_{\alpha }\left(\beta \right)$$

where λ>0 and value $P_{\alpha }\left(\beta \right)$ means the penalty. The elastic net is a mix of ridge regression (Tikhonov regularization) and LASSO (least absolute shrinkage and selection operator). For 0⩽α⩽1 penalty $P_{\alpha }\left(\beta \right)$ is a linear combination of vector norm of estimators β in spaces $L_{1}$, $L_{2}$. It is given by Equation (10):

(10) $$P_{\alpha }\left(\beta \right)=\left(1-\alpha \right)\frac{1}{2}{\left\|\beta \right\|}_{L_{2}}\;+\;\alpha {\left\|\beta \right\|}_{L_{1}}$$

This technique causes a reduction of estimators of unknown parameters. Because of this the use of the elastic net method to solve the inverse problem in tomography allows accurate and stable reconstruction images to be obtained [8].

### 2.4. Electrical Impedance Tomography

**Figure 3:**
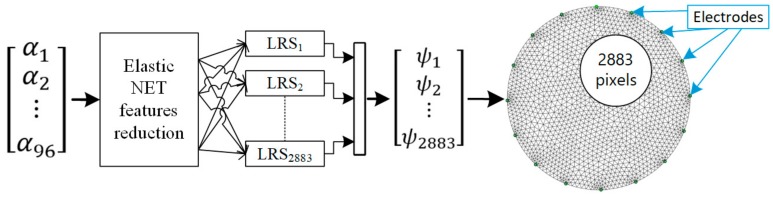
Model of the EIT system converting electrical signals into a 2D image of the cross-section.

The research described in this section uses a method based on many separately trained logistic regression subsystems. The test object was a tank filled with liquid (tap water) with a diameter of 300 mm. A total of 16 electrodes were arranged around the walls of the tank. The data was obtained through EIT. The measuring vector constituting the LRS input consists of 96 input variables (Figure 3). Each of the measurements reflects the voltage drops between 96 pairs of electrodes. In [31] a detailed description of the method of generating the measurement vector was presented. Based on 96 measurements, 2883 LRSs were trained. Each of the 2883 subsystems generates only one binary value, which is then displayed as the pixel of the output image. Since among the 96 input variables considered in the context of a singular binary output a significant number of measurements could be mutually correlated, they were reduced by an elastic net.

The selection of the number of input parameters was estimated separately for each pixel. Figure 3 shows the workflow of the EIT system, converting the input electrical measurements into the output image.

**Figure 4:**
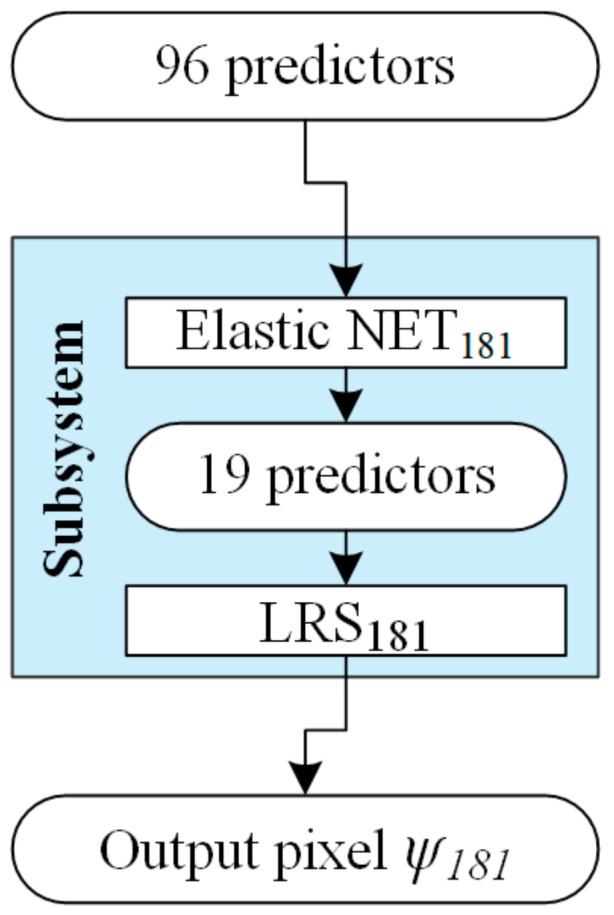
Model of elastic net + LRS hybrid subsystem dedicated to a particular pixel ψ_181_.

At the input of the EIT system, there are 96 electrical measurements. Then, thanks to the elastic net, each input vector dedicated to a specific pixel is reduced to a dozen or so elements. This situation is shown in Figure 4 where 96 predictors of pixel No. 181 (ψ_181_) are reduced to 19.

The input vectors, reduced by means of the elastic net, become the inputs of algorithmic subsystems based on the logistic regression (LRS) principle. It should be noted that we are dealing here with a hybrid system, because it combines two methods: the elastic net and logistic regression. In addition, the use of a set of separately trained LRS turns the under-completed problem into over-completed, which significantly improves the chances for better quality of reconstruction. An important issue is also the fact that machine learning in the presented method is applied in two stages. The first stage, when the number of input measures is reduced using the elastic net, and the second stage, when the inverse problem is solved with the use of multiple LRS.

To generate an appropriate training data set, a physical model of an industrial tank was built (Figure 1). Using the finite element method, the tank cross-section mesh together with the electrode system was designed using the MATLAB/EIDORS toolbox. Algorithms generating learning instances were also developed, solving the forward problem. Each case consists of a measurement vector and image generated on a two-dimensional mesh of pixels.

**Figure 5:**
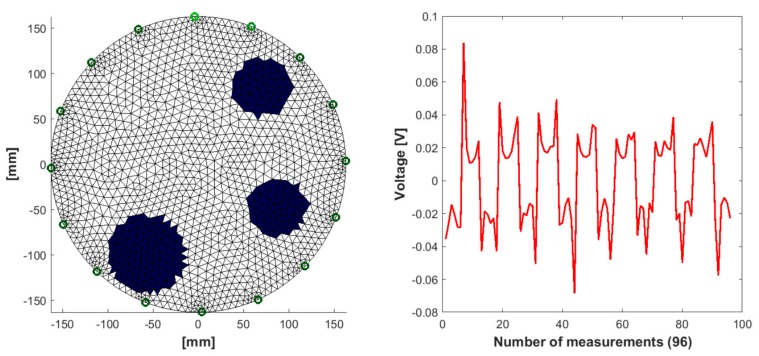
A measuring case generated with the simulation method of EIT with a graph showing the 96 voltage measurements between different pairs of electrodes.

Figure 5 shows one of the 3281 generated cases used for training a predictive system for an EIT model with 16 measuring electrodes (see Figure 3). 

Simulation training cases were generated in such a way as to take into account various (random) amounts of inclusions, diameters and positions relative to the tank wall. The presented model corresponds to a 96-element voltage measurement vector. Polarity of the electrodes changes during individual measurements. For this reason, the voltages take positive and negative values. A more precise method of generating simulation data in the form of pseudocode is presented in the article [47].

In the simulation algorithm, Gaussian noise has been implemented with a standard deviation of 4% on the value of a given measurement. The way of adding noise for measurements is presented in Algorithm 1.

**Algorithm 1** The MATLAB code to generate noise for measurements1: error_level = 0.04; % assumed 4% measurement error level 2: sigma_vector = error_level * measurements; % measurements—columnar vector with measurements % adding 4% error to the measurement values; sigma_vector—columnar vector of standard deviations 3: measurements = measurements + sigma_vector.* randn (length(measurements),1)

**Figure 6:**
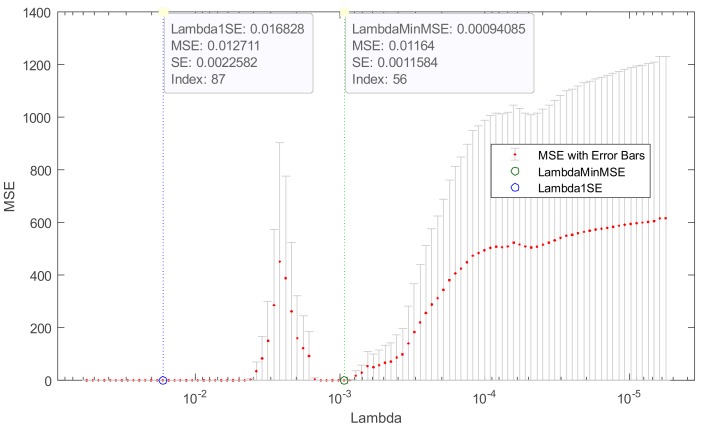
Cross-validated MSE of elastic net fit (alpha = 0.9) for pixel ψ_181_.

Figure 6 shows a cross-validated MSE (mean squared error) of the elastic net with *α* = 0.9 for an exemplary single pixel ψ_181_ of the output image. Coefficient *α* in Equation (10) is the weight of LASSO versus ridge optimization. The value *α* = 1 represents LASSO regression, *α* close to 0 approaches ridge regression, and other values represent elastic net optimization.

The figure indicates two specific lambda values marked with green and blue dashed lines. The green, dashed line shows the value of lambda with a minimum cross-validated mean squared error (LambdaMinMSE). The blue, dashed line indicates the greatest lambda that is within one standard error of the minimum MSE (Lambda1SE). This lambda value makes the sparsest model with relatively low MSE.

**Figure 7:**
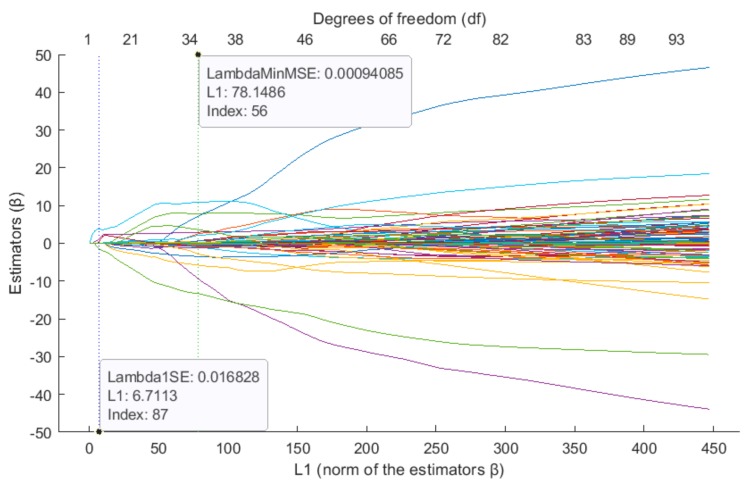
β vs. L1—trace plot of coefficients fit by elastic net (alpha = 0.9) for pixel ψ_181_.

Figure 7 on the lower horizontal axis shows the values of estimators β from the L1 norm. The horizontal axis at the top reflects the degrees of freedom (df), meaning the number of nonzero values of beta (β). Beta values represent the coefficients of a sequence of regression fits, as returned from the LASSO function. B is a (*p* × *N*) lambda matrix, where *p* is the number of predictors. Each column of β is a set of coefficients LASSO calculates using one lambda penalty value. When L1 values decrease, the number of degrees of freedom (predictors) also decreases.

**Figure 8:**
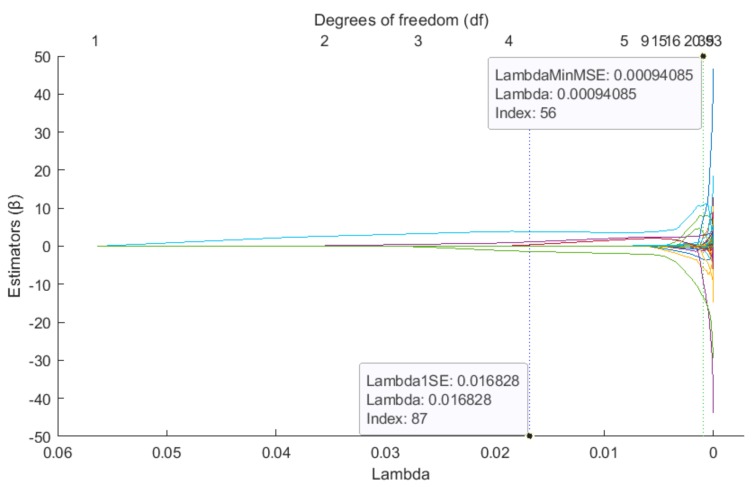
β vs. lambda—trace plot of coefficients fit by elastic net (alpha = 0.9) for pixel ψ_181_.

Figure 8 is analogous to Figure 7, however, it shows the horizontal axis reflecting the lambda coefficient in relation to the values of beta. Lambda is a penalty factor. It can be noted that along with lambda’s growth there is also a growth of the predictors’ number (df), by means of which the binary value of a given pixel can be predicted. The largest reduction of input variables takes place in the range of 0<λ<0.005. It can be seen that a slight increase in the lambda parameter above zero results in a significant reduction in degrees of freedom.

### 2.5. Ultrasound Transmission Tomography

As mentioned before, the research described in this paper uses the method based on multiple logistic regression subsystems (LRS) combined with elastic net. The measurement set constituting the LRS input vector consists of 496 measurements. Each of the measurements reflects the time taken for the sound wave to travel the distance between an individual pair of transducers. Each of the 32 transducers placed around the tank walls can both emit and receive ultrasonic signals. If there are no inclusions on the sound wave path, the time is the shortest. Before starting the measurements, the system makes a reference measurement in the environment free of hidden objects. Thanks to this, the presence of some elements disturbs (decreases) the speed of sound, thus increasing the time recorded between specific transducers. On this basis, it is possible to determine the quantity, location and size of the inclusions.

The test object is an industrial tank filled with tap water. Various sets of inclusions were immersed in the water and appropriate sound velocity measurements were made. Knowledge of the location and dimensions, as well as the number, of all inclusions corresponding to individual measurements allowed the creation of a simulation algorithm. In this way, 3602 cases of simulation measurements were generated. The simulation algorithm for generating input data for UST has been developed analogically to the EIT method (see Figure 5).

As mentioned previously, each training vector consisted of 496 measurements and one binary output image with a resolution of 128 × 128 = 16,384 pixels. The number of measurements is the result of using 32 transducers. During one measurement cycle, one of the transducers acts as the sound wave emitter. In the same time the other sensors receive the emitted signal. In this way the full matrix of input vectors counts 992 (32 × 31) measurements. It should be noted that half of the measurements concern the same transducers. The sound wave moves at the same speed regardless of the direction ($v_{1-2}=v_{2-1}$), so the measurement matrix should be symmetrical. Due to measurement errors, there are usually small differences between the measurements ($v_{1-2}\neq v_{2-1}$), hence the symmetric matrix is transformed into a triangular matrix that contains the average values of the sound waves’ speed, as shown in Equation (11):

(11) $$v_{i,j}=\left[\begin{array}{ccc} \begin{array}{c} v_{1,1}\\ v_{2,1} \end{array} & \cdots & \begin{array}{c} v_{1,31}\\ v_{2,31} \end{array}\\ \vdots & \ddots & \vdots \\ v_{32,1} & \cdots & v_{32,31} \end{array}\right]\to \left[\begin{array}{ccc} \begin{array}{c} {\bar{v}}_{1,1}\\ {\bar{v}}_{2,1} \end{array} & & \\ \vdots & \ddots & \\ {\bar{v}}_{32,1} & \cdots & {\bar{v}}_{32,31} \end{array}\right]$$

where *I* is the number of transducers, and *j* is the number of individual measures in one cycle.

### 2.6. The Method of Reconstruction

In this further part of the study, the reconstruction with application of logistic regression is presented. Based on measurements $x\in R^{m}$ obtained from sensors or transducers for the *j*-th finite element, 1⩽j⩽k, the probability of inclusion should be calculated as follows:

(12) $${\hat{y}}_{J}=\frac{e^{x{\hat{b}}_{J}}}{1\;+\;e^{x{\hat{b}}_{J}}}$$

where ${\hat{\beta }}_{J}\in R^{m}$ is the estimator of unknown parameters β for the logistic regression in Equation (2). This process should be repeated for each finite element. The result is a sequence ${\left\{{\hat{y}}_{J}\right\}}_{1\leq j\leq k}$, where ${\hat{y}}_{J}\in \left(0,1\right)$ for 1⩽j⩽k probabilities of hidden object presence for imaging domain. 

Modeling of the imaging cross-section therefore consists of the identification of these finite elements, which are a reflection of the detected inclusions and, consequently, on the display of image reconstruction. The main task for solving the problem in question therefore lies in finding a classifier that shows inclusion areas based on the sequence of probabilities. According to the sequence of probabilities of inclusion occurrence (sought for hidden objects), a sequence consisting of elements such as success and failure due to the classification threshold $l\in \left(0,1\right)$ should be defined. Success corresponds to the existence of an inclusion for a finite element. Otherwise the finite element does not consist of an inclusion.

For different thresholds 0≤l≤1 we can obtain different reconstructions of the visual field. The reconstruction of the imaging domain is the sequence $v_{rec}\left(\hat{y},l\right)={\left\{v_{j}\left(l\right)\right\}}_{1⩽j⩽k}$, where $v_{j}\left(l\right)=0$ for ${\hat{y}}_{J}<l$ and $v_{j}\left(l\right)=1$ for ${\hat{y}}_{J}⩾l$. The measure of reconstruction quality was the calculation of the value of two indicators. The first indicator determines the effectiveness of the reconstruction and the second its accuracy. To make a meaningful comparison between the pattern and the obtained image reconstruction, the basic property of the scalar product was used (Cauchy–Bunyakovsky–Schwarz inequality).

Let $v_{rec}\left(\hat{y},l\right)$ be an image reconstruction corresponding to measures $x\in R^{m}$ and let $y={\left\{y_{j}\right\}}_{1⩽j⩽k}$ be a pattern that corresponds to the same measures. We define the compatibility ratio (CR) as Equation (13):

(13) $$CR\left(l\right)=\frac{\left\langle v_{rec}\left(\hat{y},l\right),y\right\rangle }{\sqrt{\left\|v_{rec}\left(\hat{y},l\right)\|\|y\right\|}}$$

In case the sequences $v_{rec}\left(\hat{y},l\right)$ and y are collinear ($v_{rec}\left(\hat{y},l\right)=ry$, where $r\in R\setminus \left\{0\right\}$), then $CR\left(l\right)=1$.

The relative error (RE) of reconstruction is another indicator that is useful for measuring accuracy:

(14) $$RE\left(l\right)=\frac{\left\|v_{rec}\left(\hat{y},l\right)-y\right\|}{\left\|y\right\|}$$

Because the elements of vectors $v_{rec}\left(\hat{y},l\right)$ and y belong to the binary set, the above indicator shows what percentage part of the imaging domain is different from the pattern image.

## 3. Results

**Figure 9:**
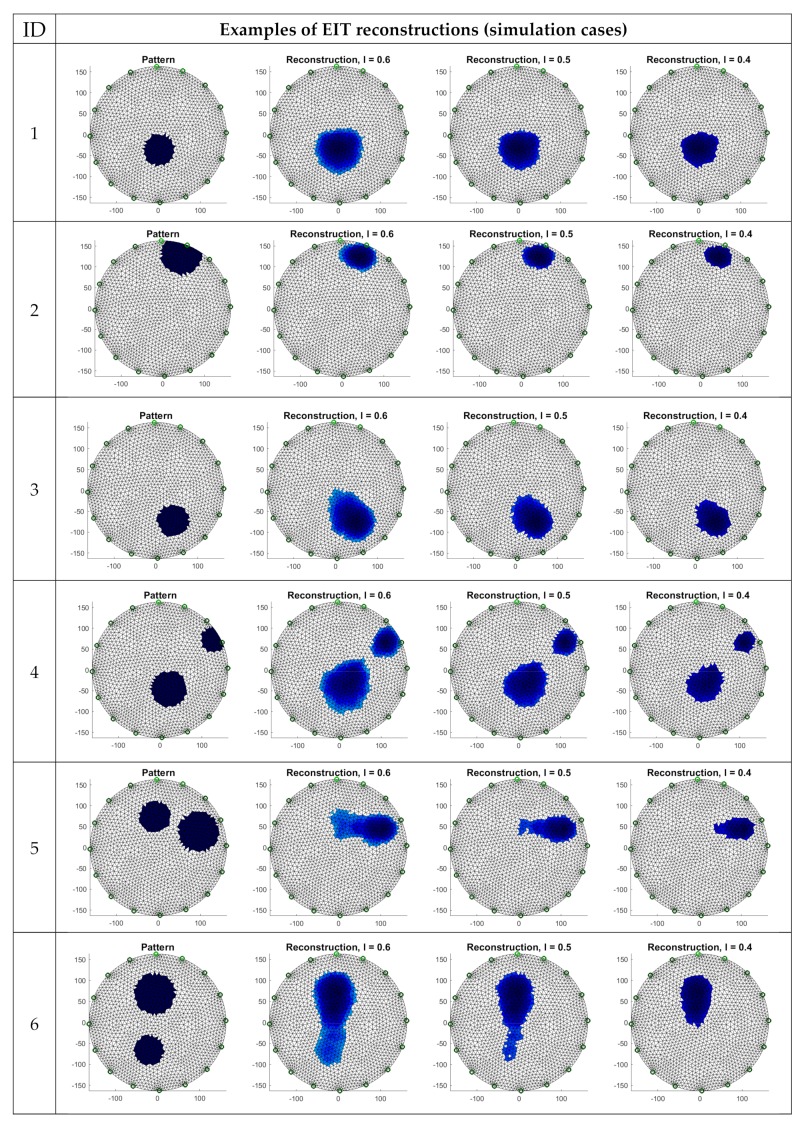
EIT image reconstructions for different classification thresholds *l.*

In order to compare cases of reconstruction of EIT and UST, data generated by simulation were used. Figure 9 shows six examples of reconstructions of EIT made using an elastic net and LRS. A pattern image was assigned to each analyzed case, and the performed reconstructions were divided into three variants differing with the applied coefficient *l* (classification threshold): *l* = 0.6, *l* = 0.5 and *l* = 0.4. 

Analyzing the obtained images, it can be noted that it is not possible to determine which level of the coefficient *l* is suitable for all reconstructions. For example, in the case of No. 1, containing a single inclusion, the best result was gained for *l* = 0.4. In turn, in the case of No. 2, despite also a single inclusion, the image closest to the pattern seems to be the image obtained using *l* = 0.6. Cases 5 and 6 with double inclusions are not satisfactory with any of the used parameters *l*.

**Figure 10:**
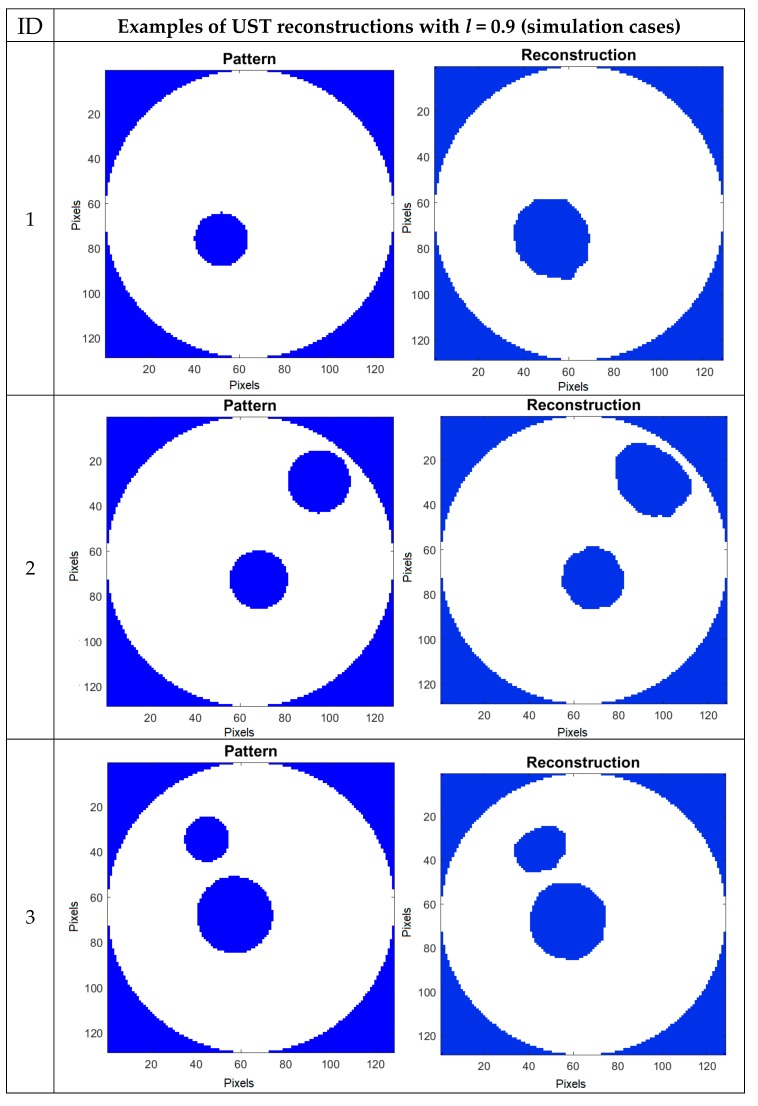

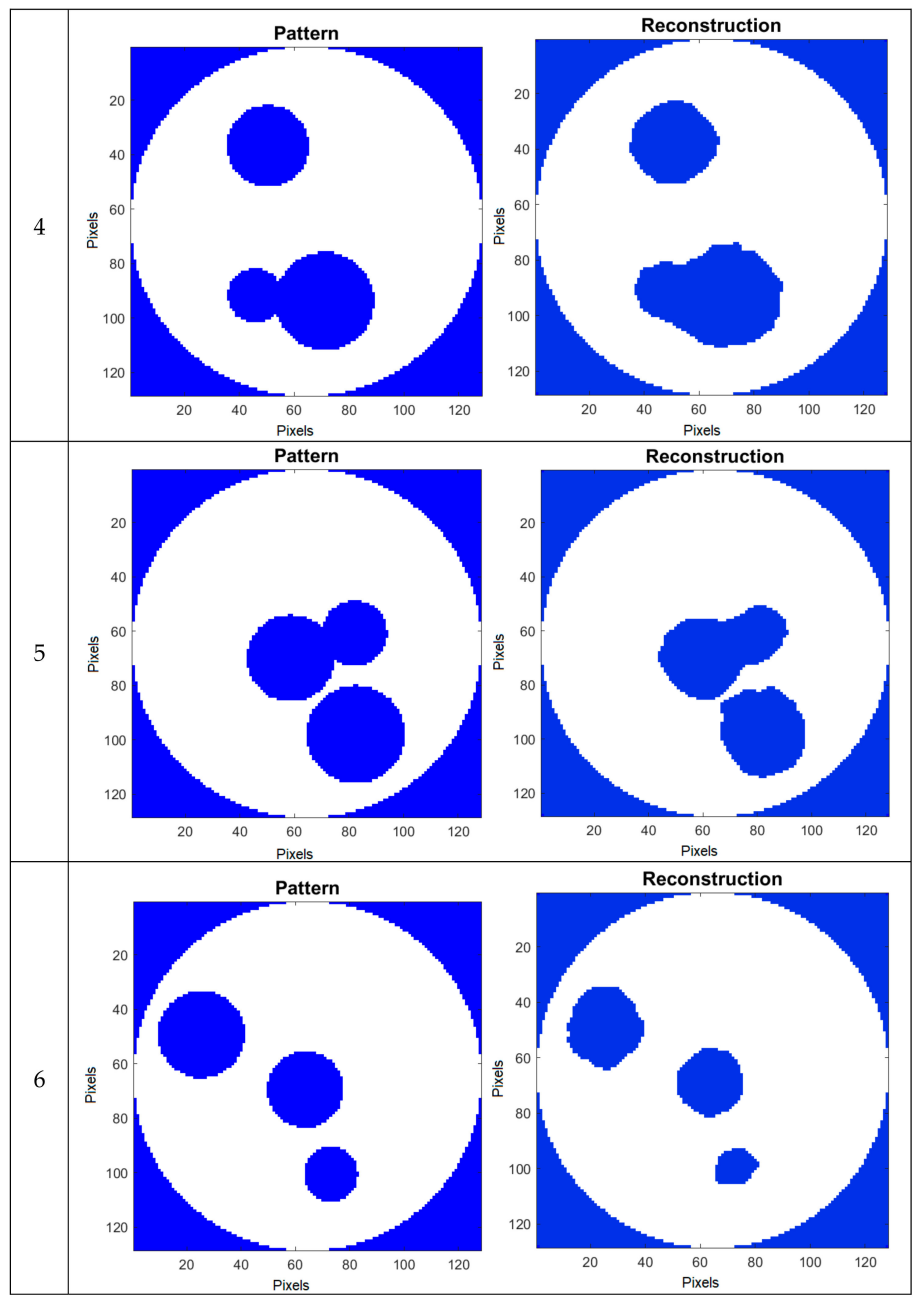
UST image reconstructions for classification threshold *l* = 0.9.

Figure 10 shows the cases of reconstruction of images obtained using an elastic net and LRS with the UST method. All presented reconstructions were made with the classification threshold *l* = 0.9. The value of coefficient *l* has been selected experimentally. The obtained images are more repeatable than in the case of EIT. In contrast to EIT, one constant value of the *l* factor, suitable for different reconstructive cases, can be selected in UST.

Table 1 and Table 2 show the values of reconstruction quality assessment coefficients. As is known, the image compared to the pattern may roughly inform about the quality of the tomography method, but only the use of objective measures enables a meaningful assessment. Table 1 presents CR and RE indices for all EIT reconstructions performed, with respect to three variants of the factor *l*. The higher the compatibility ratio (CR) and the smaller the relative error (RE), the better the quality of the reconstruction.

As mentioned in the introduction to this article, cases in which inclusions are relatively small and occur individually (not in clusters) are particularly difficult to tomographically image. One of the most commonly used measures of imaging quality in CT is RMSE (root mean squared error). This indicator is suitable for regression problems, especially where there are large inclusions or a large number of them. In the described case, the output image consists of pixels that take only binary values. For example, if there was one small inclusion in the field of view that would not have been detected by the tomograph, then the RMSE would be close to zero. To avoid such misunderstandings, it was decided to use the CR indicator, which in the mentioned case would reach the value of 1.

The expected time of reconstruction with the use of laptop (battery powered, Intel Core i5 2nd generation) for EIT was about *t*_EIT_ ≈ 10^−3^ s, and for UST *t*_UST_ ≈ 8·× 10^−3^ s. It should be taken into account that a significant part of this time was not absorbed by the calculation, but by generating a graphic image.

To enable comparison of EIT with UST, both arithmetic mean values of CR and RE coefficients are shown in both tables. It can be seen that in EIT for any of the three variants of the coefficient *l*, the mean values CR and RE do not match the UST rates. On this basis, it should be stated that better reconstructions were obtained using UST.

## 4. Conclusions

The article presents examples of the use of logistic regression supported by the elastic net for tomographic imaging in the context of two methods: EIT and UST. The novelty of the presented concept is the training of many logistic regression subsystems (LRS) operating simultaneously, thanks to which each of them generates a binary value of a single pixel of the reconstructed image. The number of LRSs is equal to the resolution of the output image. With this approach, when each LRS based on several or many hundred input variables supports one output, many predictors can be correlated with each other. This causes a distortion of the training process and increases the risk of an overfitting. To filter out unnecessary input variables and reduce the number of predictors, the elastic net method was used. Reduction of inputs significantly simplifies the tomographic system, thanks to which several thousands of LRS subsystems can be used in parallel, avoiding a long reconstruction time. The research carried out showed that the time of a single EIT reconstruction oscillated about *t*_EIT_ ≈ 10^−3^ s, and UST *t*_UST_ ≈ 8·× 10^−3^ s. This is a sufficiently short time for the described method to be used in industrial processes with significant dynamics, including flow systems.

Due to the limited volume of the text, the article presents only a small part of the obtained results. On the basis of observations of several hundred reconstructions, one can note a certain regularity. In many cases, the reconstruction of objects located in the center of the field of observation by the EIT method is slightly worse, and objects located close to the electrodes are better mapped. Perhaps this is due to the fact that the electric current in the tested environment does not propagate in straight lines. In turn, in the case of UST, slightly better results were obtained for inclusions located closer to the center of the tank, and worse in the vicinity of transducers. This problem can be the result of reflection of sound waves from the tank walls, which introduces interference noise.

The analysis of the obtained results allows one to conclude that it is not possible to choose the one, universal value of classification threshold *l* appropriate for both EIT and UST. In addition, even within EIT only, the *l* factor must be selected individually, according to specific cases of reconstruction.

A possible way to overcome the above inconveniences, leading to an increase in the effectiveness of the LRS method, would be to combine the EIT method with UST. This could require installation of both types of sensors around the tested tank: electrodes for EIT and transducers for UST. This idea requires prior solving of several technical problems related to the packing of such a large number of different sensors in close proximity, but we think that it is feasible. Therefore, future research will investigate verification of the super-hybrid method not only at the algorithmic level (LRS + elastic net) but also at the physical level (EIT + UST).

## Figures and Tables

**Table 1:** EIT imaging quality parameters.

	CR for Reconstruction with:			RE for Reconstruction with:		
Case ID	*l* = 0.6	*l* = 0.5	*l* = 0.4	*l* = 0.6	*l* = 0.5	*l* = 0.4
1	0.9900	0.9927	0.9956	0.1416	0.1207	0.0933
2	0.9868	0.9820	0.9769	0.1637	0.1919	0.2187
3	0.9899	0.9926	0.9954	0.1421	0.1212	0.0955
4	0.9799	0.9812	0.9776	0.1995	0.1931	0.2124
5	0.9679	0.9606	0.9553	0.2568	0.2876	0.3088
6	0.9807	0.9724	0.9639	0.1955	0.2361	0.2736
Arithmetic Mean	0.9825	0.9803	0.9775	0.1832	0.1918	0.2004

**Table 2:** UST imaging quality parameters for *l* = 0.9.

Case ID	1	2	3	4	5	6	Arithmetic Mean
CR	0.9874	0.9915	0.9958	0.9930	0.9878	0.9864	0.9903
RE	0.1585	0.1301	0.0919	0.1185	0.1583	0.1677	0.1375
